# Hybrid Modelling of Transarterial Chemoembolisation Therapies (TACE) for Hepatocellular Carcinoma (HCC)

**DOI:** 10.1038/s41598-020-65012-1

**Published:** 2020-06-29

**Authors:** H. Perfahl, H. V. Jain, T. Joshi, M. Horger, N. Malek, M. Bitzer, M. Reuss

**Affiliations:** 1 Stuttgart Research Center Systems Biology, University Stuttgart, Stuttgart, Germany; 2 Department of Mathematics, Florida State University, Tallahassee, Florida United States of America; 3 Department of Diagnostic and Interventional Radiology, Eberhard-Karls-University, Tübingen, Germany; 4 Department of Internal Medicine I, University Department of Medicine, Eberhard-Karls-University, Tübingen, Germany

**Keywords:** Chemotherapy, Applied mathematics

## Abstract

We extend an agent-based multiscale model of vascular tumour growth and angiogenesis to describe transarterial chemoembolisation (TACE) therapies. The model accounts for tumour and normal cells that are both nested in a vascular system that changes its structure according to tumour-related growth factors. Oxygen promotes nutrients to the tissue and determines cell proliferation or death rates. Within the extended model TACE is included as a two-step process: First, the purely mechanical influence of the embolisation therapy is modelled by a local occlusion of the tumour vasculature. There we distinguish between partial and complete responders, where parts of the vascular system are occluded for the first and the whole tumour vasculature is destroyed for the latter. In the second part of the model, drug eluding beads (DEBs) carrying the chemotherapeutic drug doxorubicin are located at destroyed vascular locations, releasing the drug over a certain time-window. Simulation results are parameterised to qualitatively reproduce clinical observations. Patients that undergo a TACE-treatment are categorised in partial and complete responders one day after the treatment. Another 90 days later reoccurance or complete response are detected by volume perfusion computer tomography (VPCT). Our simulations reveal that directly after a TACE- treatment an unstable tumour state can be observed, where regrowth and total tumour death have the same likeliness. It is argued that this short time-window is favorable for another therapeutical intervention with a less radical therapy. This procedure can shift the outcome to more effectiveness. Simulation results with an oxygen therapy within the unstable time-window demonstrate a potentially positive manipulated outcome. Finally, we conclude that our TACE model can motivate new therapeutical strategies and help clinicians analyse the intertwined relations and cross-links in tumours.

## Introduction

Hepatocellular carcinoma (HCC) is one of the leading causes of cancer-related deaths worldwide, responsible for over 600,000 deaths each year^1^. In the United States alone, the incidence of HCC has tripled over the last few decades^2^. Major risk factors for HCC include viral infections such as Hepatitis B/C, cirrhosis and nonalcoholic fatty liver disease^2^. Early stage liver cancer may be treated successfully with surgical interventions such as liver resection and transplantation^3^. Unfortunately, a majority of HCC patients are diagnosed at an advanced stage when surgery is no longer possible, and these cases are typically treated with local or systemic therapy. The high rate of cancer mortality is therefore largely attributable to treatment failure for advanced disease. Thus, there is a need to develop new therapeutic strategies that are efficacious for treating intermediate and advanced disease. Indeed, several targeted approaches are currently in various stages of development^3^.

In particular, one local treatment strategy for tumours that cannot be resected or treated by radiofrequency ablation, is transarterial embolisation (TAE)^4^. Briefly, the liver receives its blood supply from dual sources – the portal vein that supplies two thirds of the normal parenchyma, and the hepatic artery that supplies the remaining one third^5^. Significantly, HCC typically derives a majority of its blood supply from the hepatic artery^6,7^. This forms the basic rationale behind embolisation therapy. Embolisation refers to the selective occlusion of blood vessels by agents such as gelatin sponges, lipiodol or drug-eluting beads (DEBs). Prior to embolisation, an angiography is conducted to identify the artery that supplies the tumour^8^. Following successful detection of a feeding vessel, embolisation is achieved by injecting it with microparticles or DEBs. Often, TAE may be combined with chemotherapy in a process known as transareterial chemoembolisation (TACE). However, a clear benefit for the addition of a chemotherapeutic agent has not yet been established. TACE is usually administered in combination with DEBs carrying chemotherapeutic agents such as doxorubicin, cisplatin or mitomycin C^9,10^.

Although clinical trials have shown a survival benefit in patients with unresectable HCC when treated with TACE^11–13^, several challenges remain in its successful implementation. Significantly, TACE remains palliative, rather than curative, and the probability of long term survival remains poor due to local and/or regional recurrence, as well as distant metastases^3,14,15^. Further, there is a need to identify patient characteristics that can distinguish between those who respond well to TACE, versus those that show little or no response^16^. Finally, since embolisation induces high levels of intratumoral hypoxia caused by vascular occlusion, and since hypoxic HCC cells are known to be resistant to chemotherapeutic agents such as doxorubicin, it may be argued that in some cases, the lack of response to TACE represents a failure to mitigate any potential antagonism between the two types of therapy.

**Figure 1:**
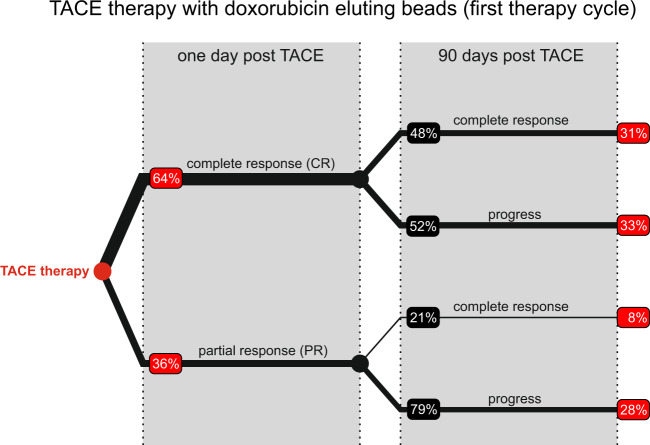
Clinical observation of TACE response. 48 liver carcinoma patients were treated with TACE, and response to treatment measured in terms of tumoral vascular perfusion compared to its value before treatment. 64% were complete responders (no residual measurable tumour perfusion) one day after the therapy. 90 days later, 48% remained complete responders while 52% showed recurrent disease. Of the partial responders (incomplete vascular occlusion with perfused tumour areas), 21% became complete responders while 79% had a progress after 90 days. (Red boxes indicate the fraction of total patients).

Indeed, in a recent observational patient study at the University Hospital in Tubingen, 48 HCC patients were treated with TACE, and outcomes observed over 90 days. The observational trial was approved by the local ethics committee (project 443/2009 BO1) and patients consented for the follow-up monitoring post-TACE. Patient response was measured in terms of changes in tumour volume perfusion CT (VPCT). Specifically, VPCT was performed 24 hours pre- and 24 hours post-TACE administration. Based on changes in VPCT compared to pre-treatment measurements, patients were classified as short-term complete responders (CR, no residual measurable tumour perfusion one day after TACE), partial responders (PR, incomplete vascular occlusion with perfused tumour areas one day after TACE) or no response (NR, no change in vascular perfusion). A third VPCT was performed 90 days post-TACE administration, where long-term responders did not show a detectable vascularised tumour. This clinical data is represented graphically in Fig. 1. Key questions arising from this study are: (1) what distinguishes short-term CR from PR; and (2) which, if any, alternative therapeutic interventions can improve the long-term response of these patients.

Our objective here is to gain a better understanding, at a mechanistic level, of the clinical data described above, and thus provide answers to the questions the data raises. We accomplish this by applying a hybrid multiscale model of vascular tumour growth that couples blood vessel formation or angiogenesis in response to a growing tumour’s nutritional demands with blood flow, nutrient transport, and the nutrient- or drug-dependent processes of cellular proliferation, quiescence and apoptosis. Our model uses as its basis, the vascular tumour growth models of Owen *et al*.^17,18^ and Perfahl *et al*.^19^. Indeed, vascular tumour growth has been extensively modeled, utilizing various frameworks such as ordinary, delay and partial differential equations, agent-based models and hybrid models that combine agent-based and continuum approaches. For instance, see^20^ for a recent review of such work. These models have been employed to study different aspects of how a growing tumour responds to therapeutic intervention, and the impact of such an intervention on the tumour microenvironment. However, relatively few models of liver cancer^21^, and none, to the best of our knowledge, of vascular HCC growth have been proposed. The model developed here is therefore the first attempt at modeling HCC growth and response to TACE in HCC.

Our modeling framework integrates multiple scales from molecular (growth factors, oxygen) to tissue level (vascularized tumour) in a hybrid framework. In particular, subcellular processes and diffusible species are simulated deterministically while cellular motion and death are stochastic in nature. We simulate the response to TACE of a cohort of virtual HCC patients, and validate our model by a qualitative comparison of our simulations to the clinical data described above. By explicitly accounting for the occlusion and remodeling of vessels – and the consequent disruption in local oxygen tension – that occurs as a result of TACE application, we gain valuable insight into what factors, at an individual patient level, are critical predictors of the success of TACE.

A key advantage of the model proposed here is that it is resolved at the level of an individual cancer cell, and a single blood vessel, which is well beyond the scope of any current imaging or measurement modality in a clinical setting. This fine level of cancer tissue resolution – coupled with the fact that basic structure of our model has been validated versus a variety of clinical data previously^17–19^ – allows us to translate model predictions into new therapeutic insights. In particular, simulations suggest that within the subset of short-term complete responders, there exists a ‘therapeutic window’ following the first application of TACE, where tumour cell numbers are at a minimum. With a view to exploiting this window, we investigate the potential of preventing new blood vessel growth during this time-frame, which should avert or delay tumour relapse.

Specifically, the major effect of embolisation is the induction of hypoxia within the tumour. In the long-term, this may be undesirable as it could lead to chemotherapeutic resistance^22^ as well as the production of angiogenic factors such as VEGF by the oxygen-starved tumour^23^. VEGF itself promotes neovascularisation and cancer cell survival^24,25^. Therefore, we hypothesize that enhancing oxygen delivery to the tumour, for instance by exposing the patient to oxygen-enriched air, in combination with TACE will improve therapeutic outcomes. Increasing systemic oxygen levels should have the added benefit of minimizing any potential antagonism between chemotherapy and embolisation, since TAE creates a hypoxic environment inducing chemotherapy-resistance in tumour cells.

The remainder of this paper is organized as follows. In Section 2 we present model simulation results, followed by a discussion in Section 3. The mathematical model and the underlying computational framework is presented in Section 4.

## Results

The multiscale model of liver cancer growth and treatment with TACE described in the model section is implemented in C++, using a GMRES solver for the PDEs, a SuperLU algorithm for resolving pressures in the vascular network, and the CVODE library for the ODEs. We remark that all simulations were carried out in a 2-dimensional domain for computational ease. However, our framework readily extends to 3-dimensions, albeit at a significant computational cost. Extensive details on how to simulate a different version of this model are available in^18^ and^19^.

### Model parametrisation

**Figure 2:**
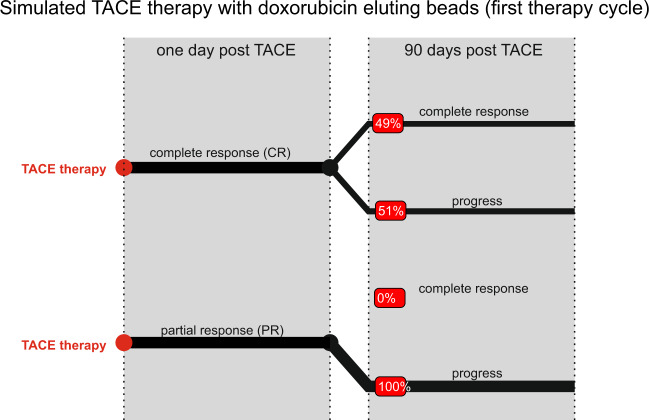
Simulation of TACE response. The simulations start by selecting either complete or partial response, by choosing the radius of influence accordingly. 49% remain complete responders after 90 days while 51% showed recurrence. In the lower branch, functional tumour vessels remain for partial responders for which all simulations lead to tumour recurrence after 90 days. Complete response after 90 days is defined by having no tumour cells in the simulation domain, while for recurrence there are tumour cells left. (Red boxes indicate the fraction of total patients).

As far as possible, model parameters were chosen from the literature. Parameters relating to the implementation of TACE were chosen so that simulations quantitatively match the clinical data shown in Fig. 1. A complete list of parameter values is provided in the section titled ‘Parameters’ in the Supplementary Information. In particular, a key parameter in our model is ${R}_{{\rm{TACE}}}$, the choice of which distinguishes between long-term partial versus long-term complete responders. The best fits are shown in Fig. 2, and the process of model parametrisation is described in further detail below.

As mentioned in the Introduction, complete responders are clinically defined as patients where no remaining vasculature can be detected by VPCT 1 day post-TACE application. In our model, this is equivalent to choosing ${R}_{{\rm{TACE}}}$ as the smallest radius so that the circle of influence of TACE covers the entire tumour. Following embolisation, the tumour is starved of oxygen (and nutrients), and can undergo a period of re-vascularisation via VEGF-mediated angiogenesis. In long-term partial responders, this re-vascularisation occurs concomitantly with tumour re-population, whereas in long-term complete responders, the tumour is eradicated due to the effect of doxorubicin. We note that doxorubicin-induced tumour cell death is a stochastic process in our model. Therefore, the parameters related to the cell death probability given by Eq. (4) are chosen to ensure that simulations match clinical data in terms of what fraction of short-term complete responders relapse in the long-term.

The failure of TACE in the short-term is simulated as follows. Clinically, patients were eligible for TACE only if their hepatic artery was identified as a major feeding vessel to the tumour, as determined by local injection with a radiological agent. If the hepatic artery was the *only* feeding vessel to the tumour, then a significant majority of such patients would be complete responders in the short-term. However, as many as 36% of the patients failed treatment within the first day (see Fig. 1). We hypothesize that these short-term partial responders are characterized by having an alternative blood supply to the tumour. In our model, this is equivalent to choosing ${R}_{{\rm{TACE}}}$ so that large enough areas of the tumour remain vascularized and unaffected by doxorubicin, released at the sites of occluded vessels by DEBs.

It should be noted that, in general, ${R}_{{\rm{TACE}}}$ should be a highly personalized parameter, taking a different value for each patient due to inter-individual variability. However, given the macroscopic nature of the available clinical data, the estimated values of ${R}_{{\rm{TACE}}}$ for complete versus partial responders may be interpreted as an average of its true – but unknown – patient-specific value.

Finally, we remark that in its current version, our model is unable to recapitulate those short-term partial responders, who go on to be complete responders 90 days post-treatment. It is possible that the tumours in such patients undergo delayed vascular collapse or that further factors, such as an induced immune response, are responsible for the delayed development of a complete response. The timing of vessel occlusion, could in turn, be a result of erratic blood flow patterns within the tumour vasculature, a level of detail beyond the scope of the model proposed here. The absence of such a mechanism, coupled with the limited diffusive length-scale of doxorubicin means that tumours in partial responders regrow in a stable manner, in our simulations.

### Patient response to TACE administration

**Figure 3:**
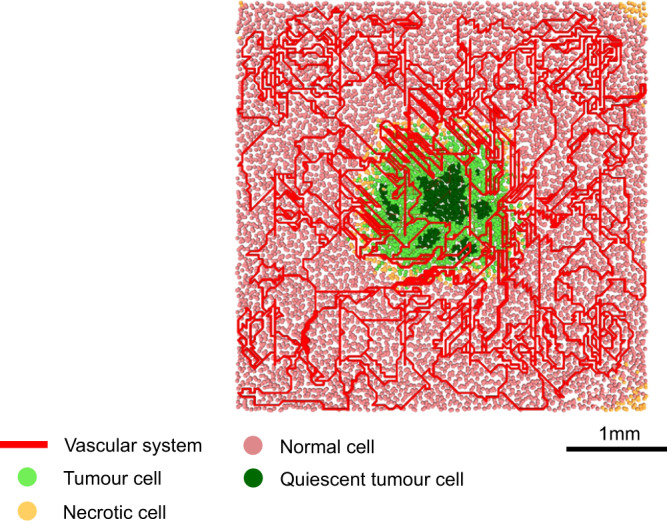
Typical tumour prior to TACE application. Healthy liver tissue is simulated until it reaches a steady state, that is the vascular network is sufficient to meet the nutritional demands of healthy cells. A tumour is then implanted in the center of the domain, and allowed to grow for 20 days, at which point TACE will be applied.

We now present the predicted response of TACE administration in our virtual HCC patients. Figure 3 shows a typical initial tumour and healthy tissue configuration for our simulations. Red lines denote the vascular network, while pink circles denote healthy cells. Tumour cells may be proliferating (light green circles) or quiescent (dark green circles) due to hypoxia. If allowed to grow unchecked, VEGF released by quiescent cells will induce further angiogenesis and the tumour will eventually take over the whole simulation domain.

**Figure 4:**
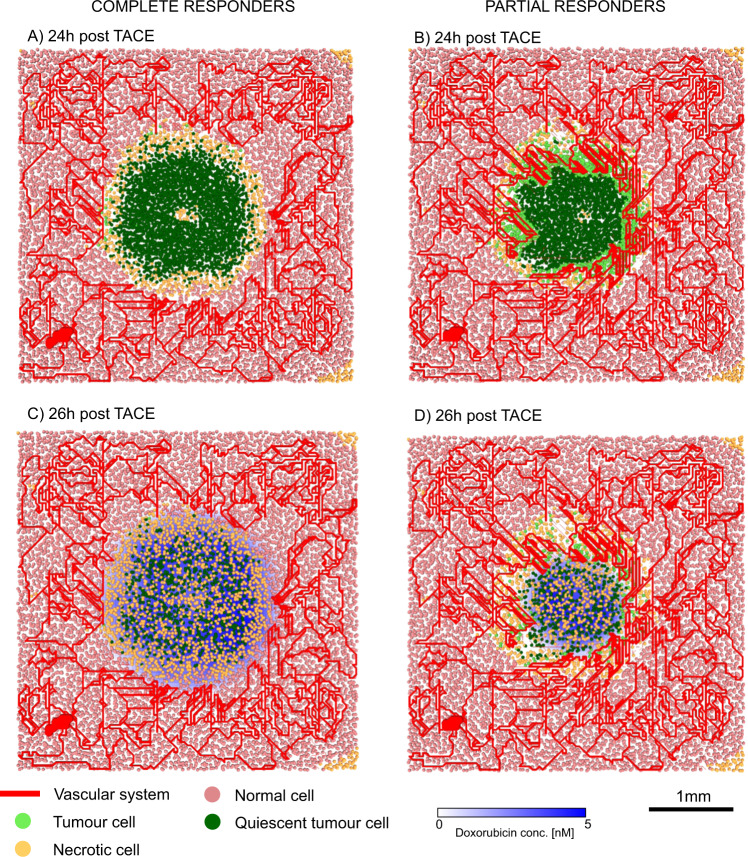
Complete and partial responders. Typical tumour and healthy tissue configuration 24 hours-post TACE application in: (**A**) complete responders and (**B**) partial responders. Typical tumour and healthy tissue configuration, with doxorubicin concentrations, 26 hours-post TACE application in: (**C**) complete responders and (**D**) partial responders.

The two possible outcomes of TACE administration are shown in Fig. 4. Figure 4A reveals that in short-term complete responders, tumour vasculature has been completely disrupted 24 hours-post therapy application, resulting in tumour cells entering quiescence. We also observe necrosis in the healthy tissue (orange circles), at the outer periphery of the tumour. This is explained by a larger-than-tumour circle of influence of TACE coupled with the fact that healthy cells are more susceptible to hypoxia than tumour cells. The effect of the slow release of doxorubicin by DEBs, located at erstwhile vessels, is shown in Fig. 4C. There is now wide-spread necrosis throughout the tumour. At this point, those patients in whom no viable tumour cells persist will go on to remain complete responders 90 days-post TACE therapy. However, even a few remaining viable tumour cells have the capacity to repopulate the former tumour region. These cells may come from avascular sanctuaries where there was little blood flow prior to TACE application, and therefore did not receive adequate levels of treatment. They may also come from those regions of the tumour where blood flow is maintained even after TACE administration, for instance, at the tumor-healthy tissue boundary.

Indeed, as can be seen in Fig. 4B, vasculature at the periphery of the tumour remains unaffected in short-term partial responders. Consequently, even though the center of the tumour is largely quiescent, cells at its boundary continue to proliferate, and are supplied with functioning vessels. Now, the slow release of doxorubicin induces a much lower degree of necrosis (Fig. 4D), and a rapid regrowth of the tumour is expected in the long-term.

### A therapeutic window and intra-tumoral oxygen enhancement therapy

In our clinical dataset, more than 50% of short-term complete responders went on to treatment failure within 3 months following TACE. Therefore, any effort to improve the long-term success of TACE must focus on this sub-population. A key advantage of our approach is that model simulations resolve tumour tissue at the level of an individual cancer cell and a single blood vessel. This knowledge of tumour dynamics both, immediately following therapy, as well as in the longer term, can be translated into new therapeutic insights.

**Figure 5:**
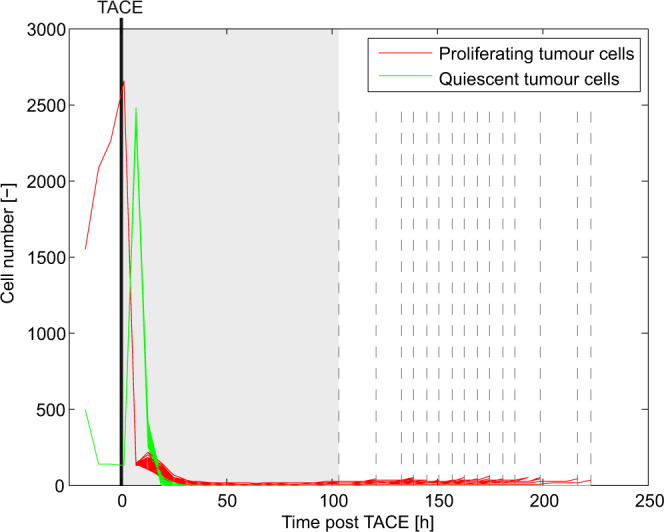
Temporal evolution of tumour cell number following TACE in 50 representative patients. TACE is applied at $t=0$ and results in the rapid necrosis of both, proliferating (red line) and quiescent (green line) tumour cells. This is followed by a period of time during which very few, or no tumour cells have survived (grey region). Those patients in whom tumour regrowth is almost certain are represented with vertical dashed grey lines, the locations of which indicate time-points at which treatment failure is predicted to occur.

With this aim, we simulate the treatment of 50 patients (short-term complete responders) with TACE, and plot tumour cell number time-courses for each patient in Fig. 5. Therapy application results in rapid large-scale necrosis followed by a period of time during which very few, or no tumour cells have survived (grey region). The slow release of doxorubicin by DEBs contributes to preventing tumour-regrowth during this period.

On a longer time-scale, the tumour will relapse provided a sufficient number of cancer cells have survived therapy. In our stochastic simulations, a minimum of 20 cells is required to re-establish the tumour with near certainty. Vertical dashed lines in Fig. 5 indicate the time-points at which treatment failure occurs in those patients in whom enough cancer cells have survived TACE. A closer look at the tumour profiles of these patients reveals a vital role for angiogenesis in ensuring tumour regrowth, as well as an explanation for the apparent randomness in times to TACE failure. Quiescent healthy cells and tumour cells within the therapy-affected regions of the tissue release VEGF under hypoxia, which results in the formation and migration of vessels into this space. Since, in short-term complete responders, the circle of influence encompasses the entire tumour, the concentration of VEGF remains high throughout this region. Consequently, sprout tip migration is largely random since a strong VEGF gradient – required for directed motion – is not established. This, coupled with the fact that surviving tumour cells require proximal vessels that supply vital nutrients and oxygen in order to re-establish the tumour, explains the observations in Fig. 5.

In fact, this inherent stochasticity in the response of the tumour immediately following TACE implies that even a small perturbation to the system could have a major impact in the long-term. We therefore hypothesize the existence of a therapeutic window within the short time-frame post-TACE application when tumour cell numbers are at a minimum (grey region in Fig. 5).

As mentioned earlier, tumour relapse only happens when enough tumour cells survive TACE, and there is efficient revascularization in their proximity. Thus, any additional treatment that prevents or delays the re-establishment of such a vascular network could have a significant impact on whether or not the tumour regrows. Given that intratumoural angiogenesis is VEGF-dependent, we investigate the potential of enhancing oxygen delivery to the tumour in order to inhibit the production of VEGF by hypoxic tumour and healthy cells. This may be achieved by exposing the patient to oxygen enriched air, resulting in intra-tumoral oxygen enhancement. Such a treatment can easily be administered in a clinical setting.

**Figure 6:**
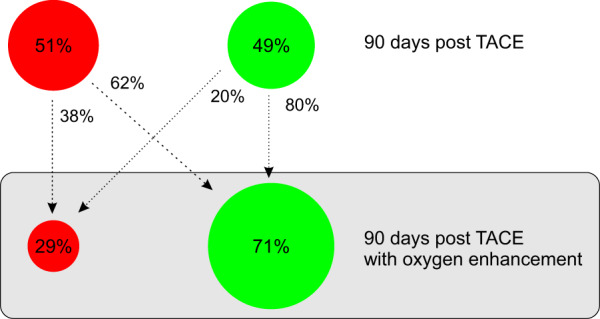
TACE combined with oxygen enhancement therapy. 51% of short-term complete responders fail therapy 90 days-post TACE administration. Combining TACE with HBO can radically improve treatment outcomes, with only 29% of short-term complete responders predicted to fail therapy 90 days-post TACE + oxygen enhancement administration. Of the patients who fail treatment within 90 days-post TACE administration, 62% become long-term complete responders if treated with TACE + oxygen enhancement. However, this combination can induce tumour relapse in a small subset of patients who were long-term complete responders when treated with TACE alone.

In our model, tumour oxygen enhancement is implemented by elevating blood oxygen concentration post-TACE by *λ*_O2_%, over a period of ${T}_{{\rm{O2}}}$ days. In the absence of data with which to estimate them, biologically realistic values were chosen for ${\lambda }_{{\rm{O2}}}$ and ${T}_{{\rm{O2}}}$. In particular, ${\lambda }_{{\rm{O2}}}$ was chosen so that such an increase in systemic oxygen is achievable simply by exposure to oxygen enriched air. The effect of administering TACE in combination with oxygen enhancement therapy is shown in Fig. 6. Under TACE alone, 51% of short-term complete responders undergo treatment failure over 90 days. However, if these patients are treated with oxygen enhancement therapy, as many as 62% of them are predicted to become long-term complete responders. Overall, the fraction of long-term partial and complete responders under TACE alone shifts from 51% and 49% respectively, to 29% and 71% respectively, under combination therapy.

Interestingly, model simulations predict the existence of sub-population within the patients who were long-term complete responders to TACE alone, for whom combining TACE with oxygen enhancement therapy is contra-indicated. Figure 6 shows that in 20% of long-term complete responders to TACE alone, oxygen enhancement therapy following TACE induces a relapse over a 90 day time period. This phenomenon can be explained as follows. The tumour tissue of this sub-population is characterized by regions where, following TACE, cancer cells would have become necrotic due to hypoxia. An increase in oxygen due to oxygen enhancement therapy now promotes cell survival and proliferation. The remaining tumour at the end of oxygen enhancement therapy is large enough to induce treatment failure over 90 days.

Thus, our model predicts that, in general, TACE followed by oxygen enhancement therapy can improve therapeutic outcomes in liver cancer patients, as compared to TACE alone. Specifically, 32% of short-term complete responders to TACE alone are predicted to benefit from the combination therapy whereas about 10% suffer from a negative impact.

### Tumour fragmentation under TACE

**Figure 7:**
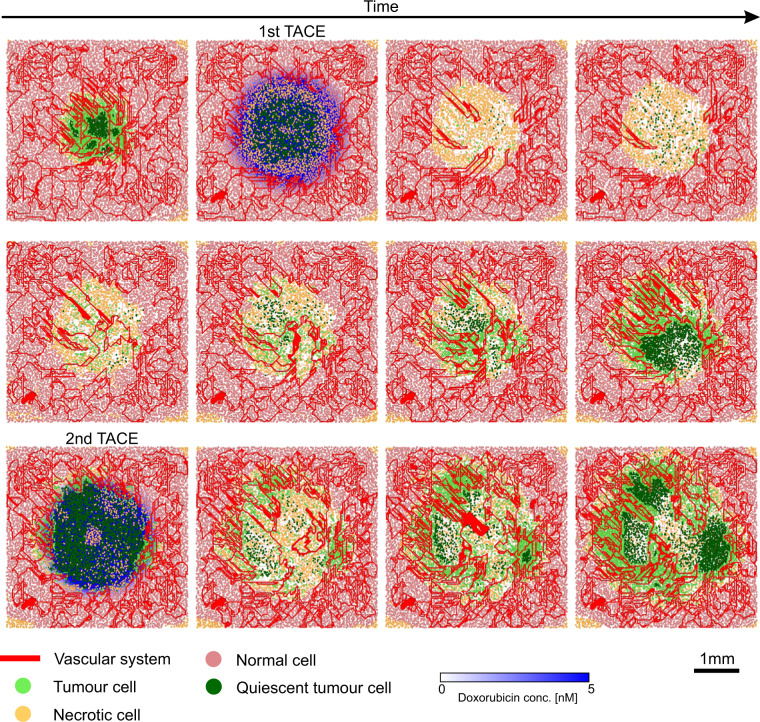
Storyboard showing two successive applications of TACE. The first therapy cycle results in a complete response 1 day post-administration (first row). The resultant hypoxic tumour cells release VEGF and induce a strong angiogenic response. Concomitantly, tumour cells proliferate in close proximity of the new vessels. The resultant tumour has invaded far into healthy tissue, and the vasculature is highly chaotic with many inter-connections (middle row). Consequently, a second round of TACE only induces a partial response. Although a large area in the tumour centre becomes necrotic, angiogenesis initiates rapidly, resulting in a clustered morphology of tumour tissue. Several areas of proliferation are observed, surrounding hypoxic cores (last row).

We finally investigate the effect of two successive TACE applications on a typical patient, who is a short-term complete responder but progresses to a long-term partial responder. A defining characteristic of such a patient in our model is the presence of viable tumour cells after the first round of TACE, which cannot be detected by VPCT. The second round of TACE is administered when blood flow is once again detected within the tumour. The predicted temporal evolution of the affected liver tissue is shown in Fig. 7.

Tumour response to the first application of TACE is as expected, with massive necrosis coupled with large-scale disruption of the vasculature. However, the hypoxic environment created due to embolisation induces new blood vessel formation, with a high degree of branching and anastomoses. This in turn promotes tumour cell proliferation, especially in proximity to the new vessels (Fig. 7, middle row). The tumour has now invaded farther into healthy tissue, and the resultant vasculature is highly chaotic, with many inter-connections. At this time, blood flow can again be detected within the treated space and TACE is administered for a second time; however, now the patient presents as a partial responder. A closer look at the tumour tissue reveals that the second round of treatment appears to have caused the tumour to fragment into several well vascularised areas with hypoxic centers (Fig. 7, last panel). We expect the resultant tumour to be nourished by more than one vessel, with potentially different sources feeding the various clusters. Such a morphology is likely to make the tumour harder to treat in the future.

## Discussion

HCC is a leading cause of cancer-related deaths worldwide, with a high mortality rate that is attributable to treatment failure for advanced disease. Over the last few decades, embolisation therapy combined with chemotherapy has emerged as a promising treatment for HCC patients. Indeed, in an observational trial at our site designed to categorise patients as short-term and long-term responders to TACE, revealed that not only did the therapy fail for ~28% of the patients, but of those that responded well to TACE initially, 52% went on to suffer a relapse within 90 days.

With a view to explicating these observations at a mechanistic level, we proposed a hybrid multi-scale model of TACE therapy application in virtual HCC patients. Our framework is an extension of a well established agent-based model of vascular tumour growth^19^. Parameters specific to TACE therapy were calibrated versus the dataset described above. Resultant model simulations were used to gain critical insight into questions such as why some patients do not respond initially to TACE and of those that do, why does the therapy eventually fail.

In particular, a key parameter in our model is the radius ${R}_{{\rm{TACE}}}$ of influence of TACE, which determines what fraction of the tumour vasculature is affected by embolisation. Simulations predict that complete response in the short-term requires the embolisation of all intra-tumoural vessels. This may be interpreted as the tumour being predominantly fed by a single vessel, the hepatic artery. On the other hand, short-term partial responders are characterized by an ${R}_{{\rm{TACE}}}$ small enough so that it leaves a fraction of tumour vessels unaffected. This may be interpreted as the tumour having more than one feeding vessel, resulting in incomplete vessel occlusion and subsequent TACE failure.

In addition to the occlusion of tumour vessels, TACE also involves the slow-release of the chemotherapeutic drug doxorubicin by DEBs located at erstwhile vessel sites. Model simulations revealed that the treatment failure over a longer time-scale in short-term complete responders occurs due to the presence of a small number of viable tumour cells that have eluded the cytotoxic effects of chemotherapy. This highlights a major strength of our modelling approach. Clinically, short-term or long-term success was interpreted macroscopically, in terms of the presence or absence of blood perfusion within the diseased liver, as detected by VPCT. In fact, no current measurement modality can detect the presence of individual cancer cells or microvessels. However, our multi-scale model provides a microscopic view of the tumour space, resolved at the level of an individual cell. We were thus able to obtain an understanding, at a functional level, of factors underpinning the long-term failure of TACE in some patients.

In fact, this fine-grained knowledge of tumour dynamics following therapy, as provided by model simulations, suggests a novel – and perhaps counter-intuitive – treatment that may preclude tumour relapse in short-term complete responders. Specifically, we propose the existence of a therapeutic window immediately following TACE application in such patients, when the tumour burden is at a minimum and the occluded vasculature has not yet started to recover. At this stage, the tumour is unstable and susceptible to small perturbations in the form of additional interventions. One such possibility is oxygen enhancement therapy, which is easily administered, for instance, by exposing patients to oxygen-enriched air. If applied during this time-frame, this treatment was predicted to prevent tumour relapse in up to 64% of patients who would otherwise have gone on to treatment failure in the long-term. We remark that here, we did not consider hyperbaric oxygen therapy (HBO) – that has been proposed as a potential treatment strategy in some cancer types^26^ – for two reasons. Firstly, even a modest increase in systemic oxygen levels was enough to induce a strong therapeutic response in our virtual HCC patients. Secondly, HBO has been associated with adverse side-effects^27^.

Finally, model simulations also revealed a potential drawback of the successive application of TACE. The hypoxic environment created by the first application of TACE was predicted to result in highly chaotic revascularisation of the tumour space, coupled with a more diffuse tumour. A second round of TACE was unable to achieve complete vascular occlusion and a clustered morphology of the tumour emerged, with several areas of proliferating cells surrounding hypoxic cores. The resultant tumour is expected to be fragmented with several blood vessels feeding it, and consequently much harder to treat.

Our model, validated by comparison against available clinical data, generated qualitatively realistic tumour tissue architecture. These simulations yielded valuable insights into the observed response to TACE and suggested complimentary treatment strategies that can benefit patients in the longer-term. However, we emphasize that multiscale models are still in their infancy and quantitative confidence in their predictions requires extensive parametrisation and validation, which must be conducted in close collaboration with experimentalists and clinicians. For instance, histopathological analysis of HCC biopsy specimens may be used to compare simulated versus real tissue. Further, new technologies such as electron paramagnetic resonance imaging can produce visualisations of oxygen levels in normal and tissues^28^ which can help in model validation. Such data can in turn, be used to refine the model. Indeed, in future iterations, we plan to integrate our model with pharmacokinetic and pharmacodynamic models that provide organ-specific concentration of a drug over time. This information would provide boundary conditions for the multiscale model, leading to a more detailed description when a combination therapy with systemic application is administered. We are also in the process of expanding the scope of the model to simulate HCC treatment with TACE in a 3-dimensional setting. One limitation of our model is that we only distinguish between the hepatic artery and portal vein, both of which supply blood to the liver, phenomenologically, via the model parameter ${R}_{{\rm{TACE}}}$. As better imaging data becomes available, we will be able to incorporate these details in a meaningful and mechanistic manner. In future iterations, we will also address a second limitation of this model, that is, we assume that all vessels within the radius of influence of TACE are occluded post-therapy. We therefore cannot simulate tumor relapse that might occur due to the presence of residual cancer cells that have survived TACE application since they were located in highly vascularised regions of the tumour where blood flow was maintained even after TACE.

A major advantage of the type of mechanistic modeling of cancer growth and treatment proposed here is that new therapies and combinations can be easily implemented in our framework, and optimal combinations predicted. For instance, combining TACE with the multikinase inhibitor sorafenib has shown conflicting and not very convincing results in some clinical trials^29,30^, but not in others^31^. Our approach could be used to elucidate the mechanisms of interactions of the two treatments at a cellular and subcellular level, which would help understand the tissue-level observations. Of course, the predictive potential of such modelling is dependent on estimating those parameters that are key determinants of the outcome for each simulated treatment. Indeed, experimental validation of such an approach requires intensive cooperation between modelers and experimentalists, as well as a model-driven experimentation approach. Nevertheless, continued modeling in this direction has the potential to lowering bench-to-bedside times for drug development, and optimisation of experimental design and administration schedules.

## Methods

The model of hepatocellular carcinoma growth and treatment presented here is based on the multiscale model of vascular growth proposed by Owen *et al*.^17,18^ and Perfahl *et al*.^19^. Specifically, the scope of these models is extended to simulate the effects of TACE application. The basic modeling framework and extensions are described in further detail below. For a step-by-step implementation (of a different version) of this model, we refer the reader to^18^ and^19^. Sample code to simulate the model described here is available on request.

The models of Owen *et al*.^17,18^ and Perfahl *et al*.^19^ are tissue-level hybrid multiscale models with integrated cell-cycles. The cellular proliferation model describes the progression of individual and healthy cells through the cell-cycle regulated by extracellular oxygen concentration. For each cell, progression through the cell-cycle is governed by a system of ordinary differential equations (ODEs) that keep track of the intracellular concentrations of molecular regulators of cell-cycle. Oxygen concentration, in turn, is governed by a partial differential equation (PDE) in which the vasculature acts as a source, and cellular consumption of oxygen as a sink. Under hypoxia, cells may enter quiescence, and secrete Vascular Endothelial Growth Factor (VEGF), a potent pro-angiogenic factor. VEGF disperses in the tissue via diffusion, therefore its concentration is also governed by a PDE, with quiescent cells acting as a source and the vasculature as a sink. The vascular network is modeled as system of connected tubes that undergoes changes due to internal and external stimuli. Specifically, new sprout formation is determined by local VEGF concentration. The sprout tips move through the simulation domain via a biased random walk, laying behind them immobile vessels. Perfused vessels arise via anastomoses, that is, when a sprout tip fuses with pre-existing perfused vasculature, or two sprout tips fuse together. The vascular system supplies vital nutrients to the tissue domain and can be used for drug delivery. For more details on the formulation and implementation of this basic model, we refer the reader to the aforementioned publications.

The above framework needs to be extended and adapted in order to simulate the application of TACE. During embolisation, small particles are injected in the hepatic artery. Following transport through the vascular system, small blood vessels are occluded^8^, resulting in oxygen deprivation and areas of necrosis within the tumour. This process is modeled as follows.

Tumour vasculature is characterized by its poor functional quality, instability, irregularity^32^. Therefore, we assume that all vessels within a tumour may be subject to occlusion due to embolisation. Specifically, we hypothesize a *circle of influence* with its center located at the center of mass of the, within which all vessels are destroyed upon TAE application. A key parameter in the model that determines the success of embolisation, is the radius ${R}_{{\rm{TACE}}}$ of this circle of influence. For a complete response, ${R}_{{\rm{TACE}}}$ is chosen such that the circle of influence wholly covers the tumour. On the other hand, for partial responders, the choice of ${R}_{{\rm{TACE}}}$ allows areas at the outer boundary of the tumour to remain vascularised. We remark that the qualitative results are not sensitive to the geometry of the area of influence. Under any geometry, complete response tumours must lie completely within this area, and partial response tumours are characterized by having some regions that remain vascularized; the area of this vascularized portion being more significant than its precise shape.

Finally, chemotherapy by doxorubicin-loaded DEBs is simulated by assuming that the beads are located at the positions of collapsed vessels, and drug-release occurs over a certain time-frame. Doxorubicin diffuses throughout the simulation domain and its concentration is governed by a PDE, with cellular uptake acting as a sink. Drug-dependent cell death or apoptosis is taken to be stochastic with an appropriately chosen probability distribution.

**Figure 8:**
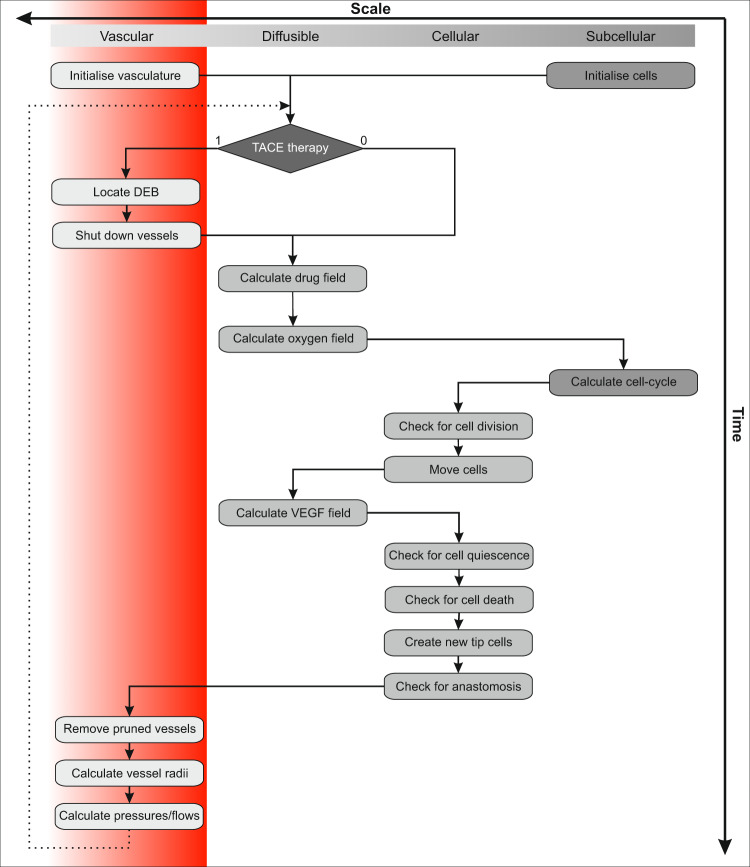
Simulation algorithm. A graphical representation of the multi-scale computational algorithm employed to simulate the multi-scale hybrid model of vascularised liver cancer growth and treatment with TACE.

A multi-scale computational algorithm is employed to simulate the model described above, integrating the ODEs operating at the subcellular level with a cellular automaton model for cell motion and proliferation, and the PDEs for diffusible substances. The vascular system overlays the tissue and is represented by a dynamic network of interconnected tubes. The various steps in implementing this algorithm are represented in Fig. 8, and described in further detail below. We remark that details on how the basic vascular growth model is simulated, are available in^19^; here, we focus on how the administration of TACE is simulated^33–38^.

The algorithm starts with the initialization of the simulation domain. Briefly, healthy liver tissue is simulated until it reaches a steady state, that is the vascular network is sufficient to meet the nutritional demands of healthy cells. A tumour (5 × 5 cellular automata tumour cells) is then implanted, and allowed to establish itself over a period of 20 days, at which point therapy is applied. In general, each time-step entails the following sub-steps. Given that oxygen diffuses on a much faster time-scale than other processes, the PDE governing its concentration is solved to steady-state. The local oxygen concentration influences intracellular cell dynamics by modulating cell-cycle times. The cell-cycle phase of each cell is updated and, where appropriate, cells can proliferate, enter quiescence or undergo apoptosis. Subsequently, the interstitial VEGF concentration is calculated – the production of which also depends on oxygen – and the vascular network updated.

The application of TACE at time ${t}_{{\rm{TACE}}}$ is simulated by occluding vessels within the predefined radius of influence ${R}_{{\rm{TACE}}}$, the choice of which has been discussed earlier. The algorithm keeps track of former vessel positions since these now act as locations of DEBs, and hence sources of doxorubicin. The PDE governing doxorubicin concentration ${c}_{{\rm{dox}}}$ is taken to be:

1 $$\frac{\partial {c}_{{\rm{dox}}}}{\partial t}={D}_{{\rm{dox}}}\Delta {c}_{{\rm{dox}}}+{\Psi }_{{\rm{dox}}}(t,x)-{k}_{{\rm{dox}}}(t,x){c}_{{\rm{dox}}}-{\delta }_{{\rm{dox}}}{c}_{{\rm{dox}}},$$

where ${D}_{{\rm{dox}}}$ is its diffusion coefficient, ${\Psi }_{{\rm{dox}}}$, the time-dependent release of doxorubicin by DEBs (see Eq. (2)), ${k}_{{\rm{dox}}}$, the cell-type dependent doxorubicin uptake rate, and ${\delta }_{{\rm{dox}}}$, its decay rate in tissue. Homogeneous Neumann boundary conditions are assumed on the simulation domain. In our numerical algorithm, Eq. (1) is discretized with a finite difference scheme and the resulting sparse linear system of equations is solved with a GMRES-solver. We remark that doxorubicin diffuses on a faster time-scale compared to the processes of cell proliferation, motion and death. Consequently, Eq. (1) is solved to steady-state within each time-step.

Since DEBs release doxorubcin over a period of time, rather than as a bolus, the rate ${\Psi }_{{\rm{dox}}}$ of its release is time-dependent and is taken to be

2 $${\Psi }_{{\rm{dox}}}(t,x)=\{\begin{array}{ll}{\psi }_{{\rm{dox}}}\,\exp (\,-\,k(t-{t}_{{\rm{TACE}}}))I(t,x) & {\rm{if}}\,{t}_{{\rm{TACE}}}\le t\le {t}_{{\rm{TACE}}}+{t}_{{\rm{dox}}}\\ 0 & {\rm{else}}\end{array},$$

where ${\psi }_{{\rm{dox}}}$ is its maximum expression rate, $(\mathrm{ln}\,2)/k$ is the half-life of doxorubicin secretion, ${t}_{{\rm{TACE}}}$ is the time at which therapy is applied, and $I(t,x)$ is an indicator function, which ensures that the DEBs are located at occluded vessel positions. We further define ${{\rm{Thr}}}_{{\rm{dox}}}$ to be the minimum concentration of doxorubicin required to elicit a cellular response. Then, the length of time ${t}_{{\rm{dox}}}$ over which the DEBs release enough drug to result in a bio-effective concentration can be computed by setting the left-hand side of the above equation equal to ${{\rm{Thr}}}_{{\rm{dox}}}$, and solving for time. That is,

3 $$\begin{array}{llll} & {{\rm{Thr}}}_{{\rm{dox}}} & = & {\psi }_{{\rm{dox}}}\,\exp (\,-\,k(({t}_{{\rm{TACE}}}+{t}_{{\rm{dox}}})-{t}_{{\rm{TACE}}}))\\ \Rightarrow  & {t}_{{\rm{dox}}} & = & \frac{1}{k}\,\mathrm{ln}\,\left(\frac{{\psi }_{{\rm{dox}}}}{{{\rm{Thr}}}_{{\rm{dox}}}}\right)\mathrm{}.\end{array}$$

At the cellular level, we also define a doxorubicin-dependent probability ${P}_{{\rm{apoptosis}}}$ of cell apoptosis as

4 $${P}_{{\rm{apoptosis}}}={p}_{{\rm{dox}}}\frac{{c}_{{\rm{dox}}}}{{p}_{{\rm{dox}},2}+{c}_{{\rm{dox}}}}\Delta t,$$

where ${p}_{{\rm{dox}}}$ is the maximum probability of cell death, ${p}_{{\rm{dox}},2}$ is the concentration of drug at which this probability is half its maximum and Δ*t* is the size of the algorithm time-step. Thus, depending on the local doxorubicin concentration, a stochastic algorithm checks whether a cell becomes apoptotic based on the above probability.

## Supplementary information

Supplementary Information.
