# Supplementary material for: Hybrid Modelling of Transarterial Chemoembolisation Therapies (TACE) for Hepatocellular Carcinoma (HCC)
Source: Sci Rep. 2020 Jun 29;10:10571. doi: 10.1038/s41598-020-65012-1 (PMC7324576; doi:10.1038/s41598-020-65012-1)
Supplement: Supplementary file 1 — Supplementary Information. [file 41598_2020_65012_MOESM1_ESM.pdf]

Appendix: Hybrid Modelling of Transarterial Chemoembolisation Therapies (TACE) for Hepatocellular Carcinoma (HCC)

H Perfahl[1,*], H V Jain[2], T Joshi[1], M Horger[3], N Malek[4], M Bitzer[4], and M Reuss[1]

[1]Stuttgart Research Center Systems Biology, University Stuttgart, Germany
[2]Department of Mathematics, Florida State University, Tallahassee, Florida, United States of America
[3]Department of Diagnostic and Interventional Radiology, Eberhard-Karls-University, Tübingen, Germany
[4]Department of Internal Medicine I, University Department of Medicine, Eberhard-Karls-University, Tübingen, Germany
[*]holger.perfahl@srcsb.uni-stuttgart.de

ABSTRACT

We extend an agent-based multiscale model of vascular tumour growth and angiogenesis to describe transarterial chemoembolisation (TACE) therapies. The model accounts for tumour and normal cells that are both nested in a vascular system that changes it's structure according to tumour-related growth factors. Oxygen promotes nutrients to the tissue and determines cell proliferation or death rates. Within the extended model TACE is included as a two-step process: First, the purely mechanical influence of the embolisation therapy is modelled by a local occlusion of the tumour vasculature. There we distinguish between partial and complete responders, where parts of the vascular system are occluded for the first and the whole tumour vasculature is destroyed for the latter. In the second part of the model, drug eluding beads (DEBs) carrying the chemotherapeutic drug doxorubicin are located at destroyed vascular locations, releasing the drug over a certain time-window.
Simulation results are parameterised to qualitatively reproduce clinical observations. Patients that undergo a TACE-treatment are categorised in partial and complete responders one day after the treatment. Another 90 days later reoccurance or complete response are detected by volume perfusion computer tomography (VPCT). Our simulations reveal that directly after a TACE treatment an unstable tumour state can be observed, where regrowth and total tumour death have the same likeliness. It is argued that this short time-window is favorable for another therapeutical intervention with a less radical therapy. This procedure can shift the outcome to more effectiveness. Simulation results with an oxygen therapy within the unstable time-window demonstrate a potentially positive manipulated outcome. Finally, we conclude that our TACE model can motivate new therapeutical strategies and help clinicians analyse the intertwined relations and cross-links in tumours.

Appendix

Model Parameters

Owen et al.[1] provides a comprehensive overview of the model parameters together with a physical description and also gives a justification for the use of dimensional and non-dimensional parameters. Here we list parameters specific for the simulations conducted in this paper.

Table 1. Parameters for the multiscale model, common to the whole simulation, to all proliferating cells, etc. (See[2–7])

Parameter	Default value	Parameter	Default value
Δt	30 min	B	0.01
Δx	40 μm	d_2	0.1 min^{-1}
Domain size	51×51	d_1	0.01 min^{-1}
$[\text{Cdh1}]_0$	0.9	$[\text{VEGF}]_0$	0.0
$[\text{cycCDK}]_0$	0.01	$[\text{p53}]_0$	0.0
M_0	5.0	k_7	0.002 min^{-1}
$[\text{p27}]_0$	0.0	k_7'	0.01 min^{-1}
$[\text{npRB}]_0$	0.0	C_{p53}	0.01
b_1	1.0 min^{-1}	k_8	0.002 min^{-1}
b_3	10.0 min^{-1}	J_5	0.04
J_3	0.04	k_8'	0.01 min^{-1}
b_4	35.0 min^{-1}	C_{VEGF}	0.01
J_4	0.04	R_{ex}	$2\Delta x$
a_4	0.04 min^{-1}		
a_2	1.0 min^{-1}	P_{O2}	3500 cm^{-1} min^{-1}
a_3	0.25 min^{-1}	P_{VEGF}	11400 cm min^{-1}
η	0.005 min^{-1}	δ_{VEGF}	10.0
M^*	10.0	D_{O2}	0.00145 cm^2/min
c_2	0.01 min^{-1}	D_{VEGF}	0.00145 cm^2/min
t_{TACE}	1320 min	R_{TACE}	0.1 cm
Thr_{dox}	0.1 nM	D_{dox}	3.0×10^{-5} cm^2/min
p_{dox}	0.003 nM/min	δ_{dox}	0.045 min^{-1}
$p_{\text{dox,2}}$	7.8 nM	Ψ_{dox}	1.0 nM/min
λ_{O2}	0.1	k	5.776×10^{-4} min^{-1}
T_{O2}	8640 min		

Table 2. Parameter values that differ for normal and cancer cells, where a dash indicates a parameter that is not defined for that cell type[2–6].

Parameter	Normal cell	Cancer cell
a_1	0.05 min^{-1}	0.4 min^{-1}
c_1	0.1 min^{-1}	0.007 min^{-1}
χ	1.0	0.0
k_8''	-0.002 min^{-1}	0.002 min^{-1}
ρ_{THR}	0.75	–
D_m	1	1
Cdh1_{THR}	0.004	0.05
$\text{cycCDK}_{\text{THR}}$	0.2	0.05
$\text{p53}_{\text{THR}}^{\text{high}}$	0.8	–
$\text{p53}_{\text{THR}}^{\text{low}}$	0.08	–
T_{death}	–	4000 min
p27_e	–	1.05
p27_l	–	1.0
V_{THR}	0.27	–
$k_{\text{VEGF}}(\mathbf{x})$	0.3	0.3
$k_{\text{dox}}(\mathbf{x})$	–	0.3

Table 3. Vasculature parameters[2–6]

Parameter	Default
P_{in}	35 mmHg
P_{out}	15 mmHg
H_{in}	0.45
μ_0	1.2 g cm min^{-2}
α	0.0
k_s	1.15 s^{-1}
k_p	1.0 s^{-1}
k_m^0	$3.\dot{3}$ s^{-1}
k_m^{VEGF}	0.0
V_0	10^{-3}
τ_{ref}	0
$\dot{Q}_{\text{ref}}$	4×10^{-5} cm^{3}min^{-1}
α_R	3.3×10^{-6}
R_{min}	1 μm
R_{max}	50 μm
τ_w^{crit}	$8.\dot{3}$ dynes cm^{-2}
T_{prune}	4000 min

Table 4. Parameters for angiogenic sprouting, or parameters that extend to endothelial cells in addition to normal and cancer cells[2–6].

Parameter	Normal	Cancer	Endothelial Cell
$k_{02}(\mathbf{x})$	13	20	5
D	0	$53.\dot{3}$ μm^2 min^{-1}	$53.\dot{3}$ μm^2 min^{-1}
γ	0	0	2×10^3 μm
N_m	1	1	2
E_m	–	–	2
$P_{\text{sprout}}^{\text{max}}$	–	–	$0.\dot{3} \times 10^{-3}$ min^{-1}
V_{sprout}	–	–	0.001
M_c	–	50	10

References

1. Owen, M. *et al.* Mathematical modeling predicts synergistic antitumor effects of combining a macrophage-based, hypoxia-targeted gene therapy with chemotherapy. *Cancer Res.* **71**, 2826 (2011).
2. Alarcón, T., Byrne, H. M. & Maini, P. K. A cellular automaton model for tumour growth in inhomogeneous environment. *J. theoretical biology* **225**, 257–274 (2003).
3. Alarcón, T., Byrne, H. & Maini, P. A multiple scale model for tumour growth. *Multiscale Model. Simul.* **3**, 440–475 (2005).
4. Alarcón, T., Owen, M. R., Byrne, H. M. & Maini, P. K. Multiscale modelling of tumour growth and therapy: the influence of vessel normalisation on chemotherapy. *Comput. Math. Methods Medicine* **7**, 85–119 (2006).
5. Betteridge, R., Owen, M. R., Byrne, H. M., Alarcón, T. & Maini, P. K. The impact of cell crowding and active cell movement in vascular tumour growth. *Networks Heterog. Media* **1**, 515–535 (2006).
6. Owen, M. R., Alarcón, T., Maini, P. K. & Byrne, H. M. Angiogenesis and vascular remodelling in normal and cancerous tissues. *J. Math. Biol.* **58**, 689–721 (2009).
7. Weinberg, B. D., Patel, R. B., Exner, A. A., Saidel, G. M. & Gao, J. Modeling doxorubicin transport to improve intratumoral drug delivery to rf ablated tumors. *J. Control. Release* **124**, 11–19 (2007).
